# Supplementary material for: Obesity Prevalence and Associated Factors in Captive Asian Elephants (Elephas maximus) in China: A Body Condition Assessment Study
Source: Animals (Basel). 2024 Dec 11;14(24):3571. doi: 10.3390/ani14243571 (PMC11672816; doi:10.3390/ani14243571)
Supplement: Supplementary file 1 [file animals-14-03571-s001.zip › animals-3325464-supplementary.pdf]

Table S1 Selection of BCS predictor variables by stepwise regression for all individuals of known age (n = 173)

	Variable	Eliminated	Number of Parameters in Reduced Models (npar)	Log-likelihood of the Given Model (logLik)	AIC	LRT	df	Pr (>χ^2)
**Random Effect**	<none>		11	-268.24	558.48			
	Facility Number	1	10	-268.98	577.96	1.4752	1	0.2245
	**Variable**	**Eliminated**	**df**	**Sum of Squares**	**RSS**	**AIC**	**F-value**	**Pr (>F)**
**Fixed Effect**	Facility Category	1	1	0.009	165.96	8.814	0.0084	0.927001
	Age	2	1	0.203	166.16	7.025	0.2017	0.653922
	Foot Disorder	3	1	1.167	167.33	6.236	1.1658	0.281831
	Daily Feed Supply	4	1	2.168	169.50	6.464	2.1641	0.143150
	Proportion of High-Calorie Feed	5	1	1.211	170.71	5.696	1.2007	0.274743
	Sex	0	1	8.976	179.69	12.561	8.8862	0.003298 **
	Outdoor Enclosure Area	0	1	32.759	203.47	34.065	32.4309	5.362e-08 ***
	Outdoor Time	0	1	22.708	193.42	25.301	22.4808	4.487e-06 ***

-Significance Code: ‘***’ ~ $p < 0.001$; ‘**’ ~ $p < 0.01$

Table S2 Selection of optimal interaction model of BCS predictor variables for all individuals of known age (n = 173)

	Number of Parameters in Reduced Models (npar)	AIC	BIC	Deviance of Log-likelihood of the Given Model (logLik Deviance)	χ^2	df	Pr ($>\chi^2$)
lmer.fit2	7	487.47	500.54	-236.73	473.47		
lmer.fit3	7	499.66	521.73	-242.83	485.66	0	0

lmer.fit2 ← lmer (BCS ~ ‘Sex’×‘Outdoor Enclosure Area’ + ‘Outdoor Time’ + (1 |‘Facility Number’),

lmer.fit3 ← lmer (BCS ~ ‘Sex’×‘Outdoor Time’ + ‘Outdoor Enclosure Area’ + (1 |‘Facility Number’),

Table S3 Selection of BCS predictor variables by stepwise regression for individuals of known age in zoos (n = 144)

	Variable	Eliminated	Number of Parameters in Reduced Models (npar)	Log-likelihood of the Given Model (logLik)	AIC	LRT	df	Pr (>χ^2)
**Random Effect**	<none>		10	-210.54	441.08			
	Facility Number	0	9	-212.80	443.60	4.5203	1	0.0335 *

	Variable	Eliminated	Sum of Squares	Mean Square	Numerator Degrees of Freedom (NumDF)	Denominator Degrees of Freedom (DenDF)	F-value	Pr (>F)
**Fixed Effect**	Age	1	0.1648	0.1648	1	135.935	0.2406	0.62457
	Foot Disorder	2	0.1678	0.1678	1	70.361	0.2475	0.62038
	Outdoor Enclosure Area	3	0.8358	0.8358	1	61.793	1.2391	0.26996
	Daily Feed Supply	4	1.0280	1.0280	1	54.429	1.5345	0.22075
	Proportion of High-Calorie Feed	5	0.7006	0.7006	1	35.520	1.0421	0.31424
	Sex	6	1.8350	1.8350	1	121.531	2.7363	0.10067
	Outdoor Time	0	8.3285	8.3285	1	22.619	12.2692	0.00195 **

-Significance Code: '**' ~ $p < 0.01$; '*' ~ $p < 0.05$
